# Esophageal temperature management during cryoballoon ablation for atrial fibrillation

**DOI:** 10.1111/jce.15724

**Published:** 2022-11-06

**Authors:** Joshua Sink, Kiran Nimmagadda, Manyun Zhao, Adin‐Cristian Andrei, Hawkins Gay, Rachel M. Kaplan, Xu Gao, Anna Pfenniger, Kaustubha D. Patil, Rishi Arora, Susan S. Kim, Alexandru B. Chicos, Albert C. Lin, Rod S. Passman, Bradley P. Knight, Nishant Verma

**Affiliations:** ^1^ Department of Internal Medicine Northwestern University Chicago Illinois USA; ^2^ Division of Gastroenterology Northwestern University Chicago Illinois USA; ^3^ Department of Preventative Medicine and Biostatistics Northwestern University Chicago Illinois USA; ^4^ Division of Cardiology Northwestern University Chicago Illinois USA

**Keywords:** atrial fibrillation, cryoablation, esophageal injury, esophageal temperature management

## Abstract

**Introduction:**

Esophageal thermal injury (ETI) is a well‐recognized complication of atrial fibrillation (AF) ablation. Previous studies have demonstrated that direct esophageal cooling reduces ETI during radiofrequency AF ablation. The purpose of this study was to evaluate the use of an esophageal warming device to prevent ETI during cryoballoon ablation (CBA) for AF.

**Methods:**

This prospective, double‐blinded study enrolled 42 patients with symptomatic AF undergoing CBA. Patients were randomized to the treatment group with esophageal warming (42°C) using recirculated water through a multilumen, silicone tube inserted into the esophagus (EnsoETM®; Attune Medical) (WRM) or the control group with a luminal single‐electrode esophageal temperature monitoring probe (LET). Patients underwent upper endoscopy esophagogastroduodenoscopy (EGD) the following day. ETI was classified into four grades.

**Results:**

Baseline patient characteristics were similar between groups. Procedural characteristics including number of freezes, total freeze time, early freeze terminations, coldest balloon temperature, procedure duration, posterior wall ablation, and proton pump inhibitor and transesophageal echocardiogram use before procedure were not different between groups. The EGD was completed in 40/42 patients. There was significantly more ETI in the WRM group compared to the LET group (*n* = 8 [38%] vs. *n* = 1 [5%], *p* = 0.02). All ETI lesions were grade 1 (erythema) or 2 (superficial ulceration). Total freeze time in the left inferior pulmonary vein was predictive of ETI (360 vs. 300 s, *p* = 0.03).

**Conclusion:**

Use of a luminal heat exchange tube for esophageal warming during CBA for AF was paradoxically associated with a higher risk of ETI.

## 1 BACKGROUND

Catheter ablation with pulmonary vein isolation (PVI) is commonly used to treat medically‐refractory, symptomatic atrial fibrillation (AF).^1^, ^2^ Atrio‐esophageal fistula (AEF) is a rare but well‐recognized complication of PVI and posterior left atrial ablation and has been reported both with radiofrequency (RF) energy and cryoballoon ablation (CBA).^2^ Though the incidence of AEF is low, with rates reported between 0.02% and 0.11%, the result is often fatal.^2^ Endoscopy has been used to evaluate patients with potential esophageal symptoms within a few weeks after catheter ablation for AF.^3^ Endoscopic visualization of esophageal thermal injury (ETI) is considered a surrogate endpoint when investigating the safety of catheter ablation technology, since studies have shown that even early‐stage ETI lesions can progress to AEF.^3^, ^4^, ^5^, ^6^ Current strategies to reduce the risk of esophageal injury include delivery of lower energy along the posterior left atrium, shorter duration applications, avoiding lesions close to the esophagus based on esophageal imaging and localization, mechanical displacement of the esophagus, and the use of a luminal esophageal temperature (LET) monitoring.^7^, ^8^, ^9^, ^10^, ^11^, ^12^, ^13^

**Figure 1:**
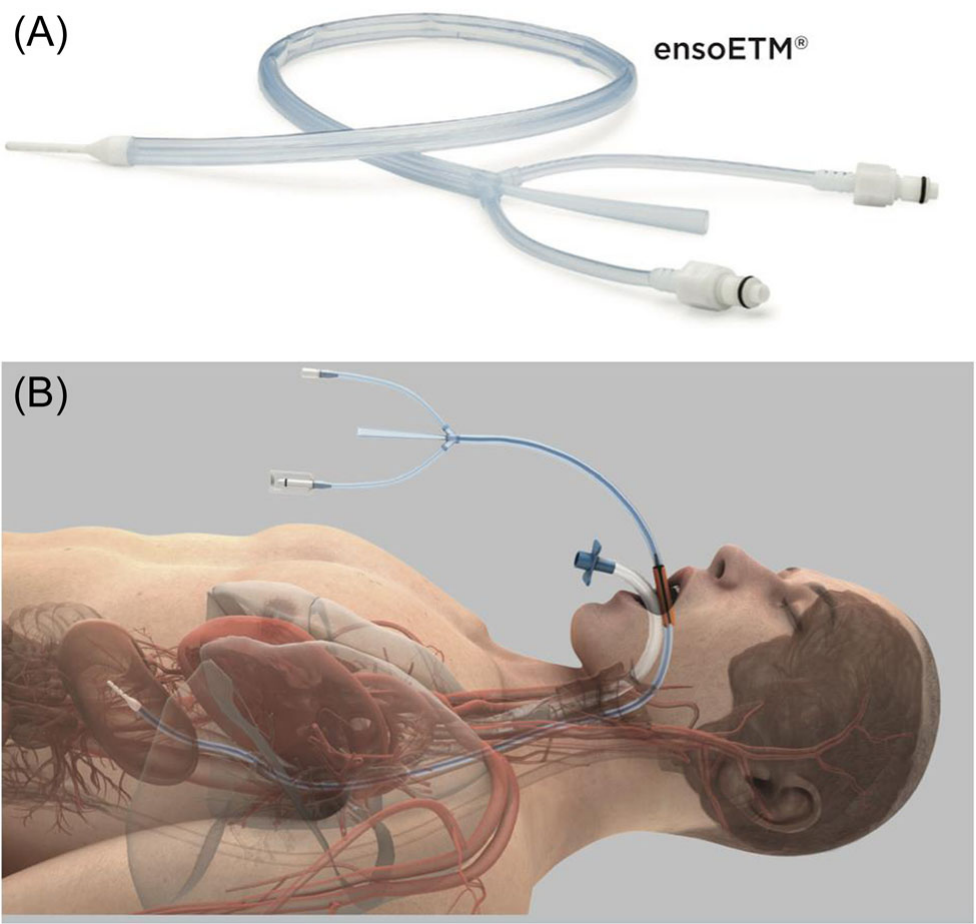
(A) Image of the EnsoETM®, demonstrating the multi‐lumen silicone tube.^14^ (B) Visualization of the esophageal heat‐exchange tube placement in the esophagus after intubation.^14^ The radiopaque tip is positioned in the stomach. The tube is connected to an external heat exchanger to adjust the temperature of recirculated water, allowing for esophageal warming or cooling.

The EnsoETM® esophageal warming and cooling device (Attune Medical) is FDA approved for esophageal temperature management. It consists of a multilumen silicone tube inserted into the esophagus and connected to an external heat exchanger to adjust the temperature of recirculated water, allowing for esophageal warming or cooling (Figure 1). The radiopaque tip is placed below the diaphragm on fluoroscopy and the tube spans the entire length of the esophagus for the duration of the case. Studies have demonstrated that esophageal cooling with this device reduces ETI without reducing ablation effectiveness during RF ablation for AF.^3^, ^4^, ^15^ The purpose of this pilot study is to evaluate the safety and feasibility of esophageal warming with the esophageal heat‐exchange tube to prevent ETI during CBA for AF.

## 2 METHODS

### 2.1 Study design and population

This prospective, randomized, double blinded, single‐center pilot study enrolled patients with refractory AF undergoing a first‐time CBA PVI procedure between October 2019 and September 2021. Forty‐two patients were randomized in a 1:1 fashion, with 23 patients randomized to undergo CBA with esophageal warming using the esophageal heat‐exchange tube (WRM) and the other 19 patients randomized to the control group of CBA with traditional LET monitoring. All patients signed an informed consent, and the study was approved by the Northwestern University Institutional Review Board.

### 2.2 Cryoballoon procedure workflow

Cardiac magnetic resonance imaging was obtained for all patients for the purpose of left atrial anatomic evaluation. All procedures were performed under general anesthesia. Standard technique was used to perform ablation with cryoballoon (Medtronic).^16^ Two‐to‐three‐min lesions were used in each vein. Additional posterior wall ablation with the cryoballoon was performed in a small subset of patients when the goal was left atrial posterior wall isolation.

### 2.3 Treatment group (WRM)

**Figure 2:**
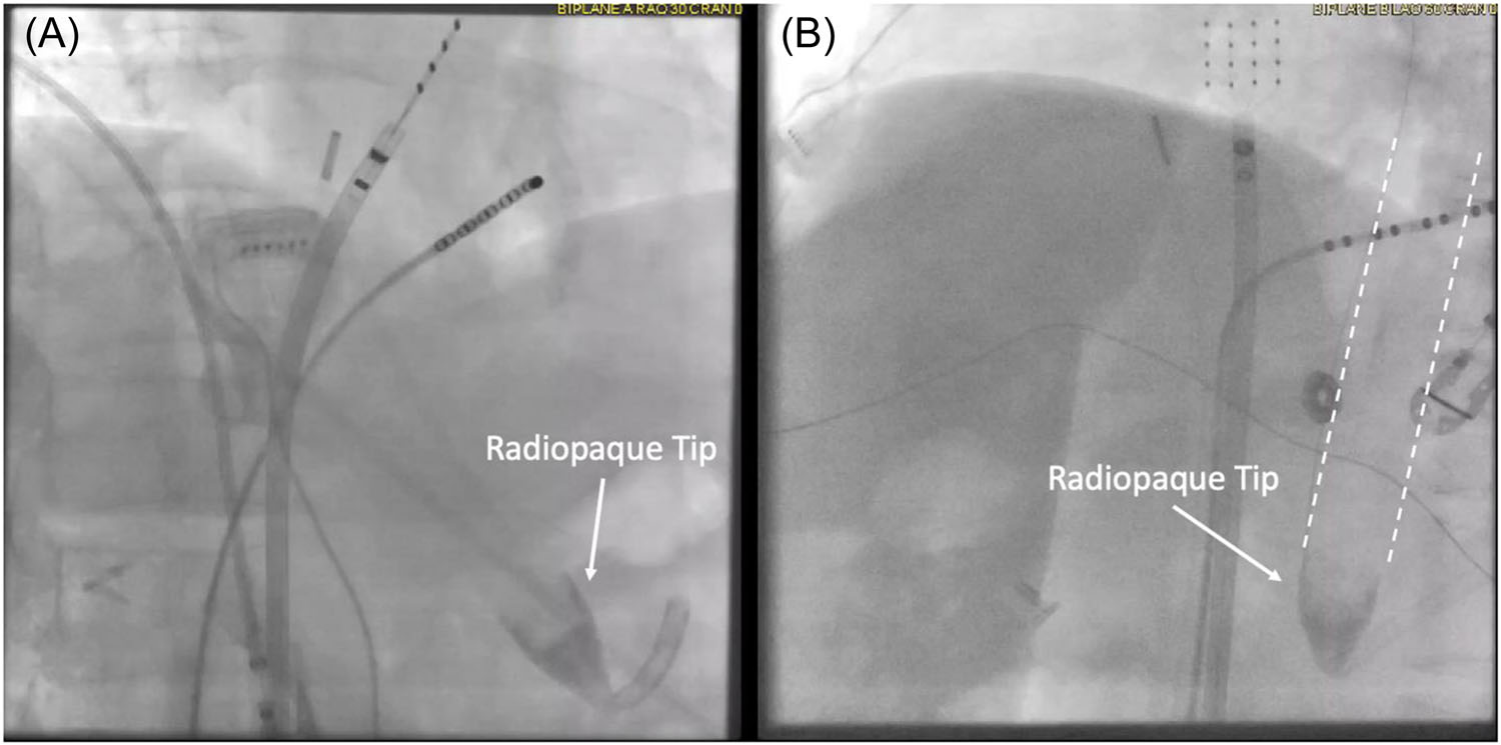
(A) RAO fluoroscopic view of the esophageal heat‐exchange tube placement. The radiopaque tip can be seen in the stomach below the diaphragm. (B) LAO fluoroscopic view of the esophageal heat‐exchange tube placement.

Patients randomized to the WRM group had the esophageal heat‐exchange tube placed by an anesthesiologist following endotracheal intubation. Placement of the device was confirmed with fluoroscopic visualization of the radiopaque distal tip below the diaphragm (Figure 2). Modulation and control of the temperature was achieved by connecting the EnsoETM® to a Blanketrol III® (Gentherm Medicine) external heat exchanger. The device was programmed to circulate warmed, distilled water at 42°C for the extent of the ablation procedure. LET monitoring was not performed in this group due to the risk of perforation of the heat‐exchange tube by the temperature probe. Core body temperature was instead monitored using a standard nasopharyngeal temperature probe in the WRM group.

### 2.4 Control group (LET)

Patients randomized to the LET group had LET monitoring performed using an Esophageal Stethoscope Temperature Sensor® (Smiths Medical), a single thermocouple temperature probe, which was repositioned as necessary to maintain proximity with the cryoballoon. The single thermocouple temperature probe is the standard LET monitoring device used during CBA at the study institution. Freezing sessions were stopped prematurely if the temperature dropped below 25°C. The esophageal heat‐exchange tube was not used in this group.

### 2.5 Endoscopic assessment

Forty of 42 patients underwent esophagogastroduodenoscopy (EGD) one day following ablation. The EGD was performed utilizing a standardized protocol. All EGDs were interpreted by a single, experienced gastroenterologist who was blinded to the patient's study group. ETI was classified into four grades: (1) erythema (2) superficial ulceration (3) deep ulceration (4) fistula/perforation. Endoscopic evidence of gastroparesis was also documented. This was defined by the presence of food in the stomach despite overnight fasting. Prophylactic use of a proton pump inhibitor (PPI) was limited and operator dependent (Table 2). However, all patients with esophageal injury identified on EGD were treated with a PPI for 30 days.

### 2.6 Follow‐up for adverse events and AF recurrence

Patients had standard of care follow‐up visits at 3, 6, and 12 months after the procedure. Patients were assessed for adverse events and AF recurrence. A combination of rhythm monitoring devices was used at follow‐up to assess for AF recurrence including extended Holter monitoring and implantable device‐based rhythm assessment. All patients were screened for new gastrointestinal symptoms at follow‐up.

### 2.7 Statistical analysis

Data summaries included means/standard deviations, medians/interquartile ranges, counts and percentages. Comparisons between the WRM and the LET groups were based on two‐sample *t*‐tests with equal variances, Wilcoxon's rank sum test, *χ* ^2^, or Fisher's exact test (if counts < 5). Two‐sided *p* Values < 0.05 were considered statistically significant and no multiplicity adjustments were made.

## 3 RESULTS

### 3.1 Baseline patient characteristics

Forty‐two patients were enrolled in the study and underwent CBA, with 23 patients randomized to the WRM group and 19 patients randomized to the LET group. Forty out of 42 patients underwent EGD 1 day after ablation—21 in the WRM group and 19 in the LET group. The two patients who did not undergo EGD withdrew because they did not want to undergo sedation a second day in a row. Baseline clinical characteristics were similar between groups, as shown in Table 1.

**Table 1:** Baseline characteristics^a^

Characteristic	Entire cohort (*N* = 42)	LET (*N* = 19)	WRM (*N* = 23)	*p* Value
Age—mean (SD)	65.6 ± 9.8	66.6 ± 8.4	64.7 ± 11.0	0.54
Female—No. (%)	9 (21%)	3 (16%)	6 (26%)	0.48
Clinical Comorbidities—No. (%)				
CHF	9 (21%)	5 (26%)	4 (17%)	0.71
CAD	3 (7%)	1 (5%)	2 (9%)	1
CVA	5 (12%)	2 (11%)	3 (13%)	1
HTN	25 (60%)	13 (68%)	12 (52%)	0.35
COPD	1 (2%)	1 (5%)	0 (0%)	0.45
DM II	3 (7%)	1 (5%)	2 (9%)	1
Paroxysmal AF	25 (60%)	11 (58%)	14 (61%)	1
Persistent AF	17 (41%)	8 (42%)	9 (39%)	1

Abbreviations: AF, atrial fibrillation; CAD, coronary artery disease; CHF, congestive heart failure; COPD, chronic obstructive pulmonary disease; CVA, cerebrovascular accident; DM II, type 2 diabetes; HTN, hypertension.

^a^ Age calculated by *t*‐test. All binary variables were tested by Fisher Exact.

### 3.2 Procedural characteristics

Procedural characteristics including number of freezes delivered, total freeze time, number of early freeze terminations, coldest balloon temperature, procedure duration, posterior wall ablation, and PPI and transesophageal echocardiogram (TEE) use before procedure were not different between groups (Table 2). When examined by pulmonary vein, there were still no statistically significant differences in procedural characteristics between groups, but there was a trend towards more early freeze terminations in the right inferior pulmonary vein (RIPV) in the LET group that was not statistically significant (47% vs. 17% with 1 or more early freeze terminations, *p* = 0.07).

**Table 2:** Procedural characteristics^a^

Characteristic	Entire Cohort (*N* = 42)	LET (*N* = 19)	WRM (*N* = 23)	*p* Value
Total # of freezes—median (IQR)	8 (7−9)	8 (7−9)	8 (8−9)	0.4
Total freeze time (s)—median (IQR)	1239 (1100−1410)	1260 (973−1525)	1228 (1115−1406)	0.93
LSPV freeze time (s)—median (IQR)	300 (300−360)	300 (300−360)	300 (264−360)	0.61
LIPV freeze time (s)—median (IQR)	300 (260−360)	300 (240−360)	300 (300−360)	0.27
RSPV freeze time (s)—median (IQR)	300 (230−360)	324 (187−360)	270 (230−336)	0.26
RIPV freeze time (s)—median (IQR)	300 (257−360)	300 (180−360)	300 (300−360)	0.61
Coldest freeze temperature—median (IQR)	−55 (−56 to −53)	−55 (−57 to −52)	−55 (−56 to −53)	1
Procedural time (min)—median (IQR)	136 (112−163)	140 (122−184)	116 (108−152)	0.31
Total # times freeze stopped early—median (IQR)	1 (0−2.0)	1 (0−2.0)	1 (0−2.0)	0.53
Use of TEE—No. (%)	26 (62%)	12 (63%)	14 (61%)	1
Posterior Wall Ablation—No. (%)	3 (7%)	2 (11%)	1 (4%)	0.58
PPI use before procedure—No. (%)	13 (31%)	7 (37%)	6 (26%)	0.52

Abbreviations: LIPV, left inferior pulmonary vein; LSPV, left superior pulmonary vein; PPI, proton pump inhibitor; RIPV, right inferior pulmonary vein; RSPV, right superior pulmonary vein; TEE, transesophageal echocardiogram.

^a^ All continuous variables shown with calculated median, tested by Wilcoxon rank‐sum test. All binary variables were tested by Fisher Exact. Temperatures shown in Celsius.

### 3.3 ETI

**Figure 3:**
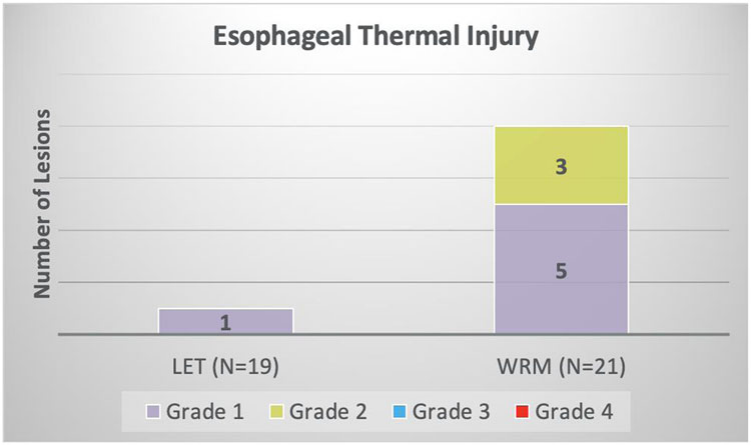
A comparison of esophageal thermal injury between the two groups. Overall, there was more esophageal injury in the WRM group compared to the LET group (*n* = 8 [38%] vs. *n* = 1 [5%], *p* = 0.02). ETI was classified into four grades: (1) erythema (2) superficial ulceration (3) deep ulceration (4) fistula/perforation. ETI, Esophageal thermal injury; LET, luminal esophageal temperature.

Overall, there were more patients with ETI in the WRM group compared to the LET group (*n* = 8 [38%] vs. *n* = 1 [5%], *p* = 0.02, Figure 3). All ETI lesions were mild to moderate (grade 1 or 2). No patient had more than one lesion. There were no grade 3 or 4 lesions identified. There were no instances of AEF or any other GI complications on follow‐up. Within the WRM group, 5/8 (63%) of lesions were grade 1, with the remaining 3/8 (38%) lesions being grade 2. In the LET group, the single lesion identified was grade 1. EGD evidence of gastroparesis was similar between groups (63% vs. 57%, *p* = 0.76).

### 3.4 Factors associated with injury

Secondary analysis was performed to identify possible factors associated with ETI in univariate fashion (Table 3). Median total freeze time in the left inferior pulmonary vein (LIPV) was found to be associated with the occurrence of ETI (median 360 vs. 300 s, *p* = 0.03), and so was procedure duration (median 146 vs. 125 min, *p* = 0.02). The number of freezes delivered, number of early freeze terminations, coldest balloon temperature, and PPI use before procedure were not found to have a statistically significant association with the occurrence of ETI. However, there was more ETI with colder balloon temperatures in the left superior pulmonary vein (LSPV) (−53.6°C vs. −50.7°C, *p* = 0.06), but the difference was not statistically significant.

**Table 3:** Univariate factors in relationship to esophageal injury^a^

Characteristic	Entire Cohort (*N* = 40)	No Esophageal Injury (*N* = 31)	Esophageal Injury (*N* = 9)	*p* Value
Number of Freezes—median (IQR)	8 (7−9)	8 (7−9)	8 (8−12)	0.45
Total Freeze Time (s)—median (IQR)	1239 (1108−1463)	1260 (1100−1410)	1209 (1195−1516)	0.78
LSPV freeze time (s)—median (IQR)	300 (300−360)	300 (291−360)	300 (300−360)	0.67
LIPV freeze time (s)—median (IQR)	300 (265−360)	300 (240−321)	360 (309−360)	0.03
RSPV freeze time (s)—median (IQR)	300 (220−360)	300 (187−360)	270 (340−336)	0.94
RIPV freeze time (s)—median (IQR)	300 (257−360)	337 (257−360)	300 (257−300)	0.15
Procedure Time (min)—median (IQR)	139 (112−165)	125 (110−152)	146 (141−211)	0.02
Early Freeze Terminations—median (IQR)	1 (0−2)	1 (0−2)	1 (0−2)	0.76
Coldest Balloon Temperature—mean (SD)	−54.6 ± 2.9	−54.5 ± 3.1	−55 ± 2.2	0.62
Posterior Wall Ablation—No. (%)	3 (8%)	2 (7%)	1 (11%)	0.55
PPI use before procedure—No. (%)	12 (30%)	10 (32%)	2 (22%)	0.7

Abbreviations: LIPV, left inferior pulmonary vein; LSPV, left superior pulmonary vein; PPI, proton pump inhibitor; RIPV, right inferior pulmonary vein; RSPV, right superior pulmonary vein; SD, standard deviation.

^a^ Coldest balloon temperature (in Celsius) calculated by *t*‐test. All other continuous variables shown with calculated median, tested by Wilcoxon rank‐sum test. All binary variables were tested by Fisher Exact.

### 3.5 Follow‐up of adverse events and AF recurrence

Patients were assessed for AF recurrence and adverse events up to 12 months out from their procedure. Overall freedom from AF recurrence was 83% in the WRM group compared to 81% in the LET group (*p* = 1.0). There were no adverse events found at follow‐up, and notably no fistula or perforation events were identified. There were no persistent GI symptoms or clinical evidence of gastroparesis at follow‐up. No patient in the esophageal warming group had a clinically meaningful increase in total body temperature during the procedure, as measured by nasopharyngeal temperature probe.

## 4 DISCUSSION

### 4.1 Main findings

This is the first prospective, randomized study to evaluate the use of esophageal warming with an esophageal heat‐exchange tube during CBA for AF. The primary finding of this study was that use of the esophageal heat‐exchange tube was paradoxically associated with a statistically significant higher risk of ETI. Previous studies have demonstrated that esophageal cooling with the esophageal heat‐exchange tube can reduce risk of ETI when used during RF ablation for AF.^3^, ^4^ Our study showed that use of the esophageal heat‐exchange tube for warming during CBA led to higher rates of grade 1 and grade 2 ETI. This finding may not accurately predict progression to AEF, though prior studies have established that mild grades of ETI can progress to AEF in the days to weeks after the procedure.^5^, ^6^

### 4.2 Effect of esophageal warming during CBA

A possible explanation for the findings of the present study is that the inflation of the esophageal heat‐exchange tube may bring the esophagus closer in anatomic proximity to the left atrium for the duration of each CBA lesion. Sarairah et al. have previously identified an increased risk of ETI with decreased distance between the esophagus and left atrium.^17^ In addition, the amount of warming (42°C) provided by the esophageal heat‐exchange tube may simply not be enough heat to overcome the cold temperatures of CBA (up to −60°C). Also, CBA lesions tend to be longer in duration than RF lesions. These differences might explain why the EnsoETM® device has been found to be beneficial for reducing ETI with RF ablation but not with CBA. Though there were no statistically significant differences in procedural characteristics, there was a trend towards increased early freeze terminations in the RIPV in the LET group. It is possible that the use of LET monitoring in the control group led to a more cautious ablation approach with an increased propensity for early freeze terminations in the setting of cold balloon temperatures. A combination of these factors may have contributed to the results. It is possible that the heat from the esophageal heat‐exchange tube itself or the preprocedural TEE caused the observed ETI. This is unlikely as all the lesions found were discrete in size and location, consistent with that expected from ablation during PVI. In addition, the injury pattern did not extend the length of the esophagus, all of which is in contact with the tube.

For the purpose of this study, a warming temperature of 42°C was used. This temperature was chosen, in part, because it is the maximum nominal temperature available to be set by the device. Unfortunately, there are not any bench or animal model studies available comparing the thermodynamic properties of different temperature settings or flow rates with this device in the setting of CBA. It is possible that different programmed parameters with this device may lead to a different result. Further studies would be necessary to evaluate this. In addition, it is not clear if the findings from the present study are applicable to other esophageal warming devices used during other catheter ablation procedures that involve cryothermal energy.

### 4.3 Factors associated with ETI

A secondary goal of this study was to identify factors associated with ETI. Previous studies have identified obesity and narrow atrial‐esophageal distance as risk factors for ETI.^17^ A time‐to‐isolation guided protocol for CBA has also been found to reduce ETI.^18^ Interestingly, the present study found that increased total freeze time in the LIPV lead to higher risk of ETI. Increased procedure duration was also associated with increased ETI, though this is likely an indirect relationship that is mediated by factors such as the increased freeze time in the LIPV. No other clinically relevant portions of the procedure were identified to be prolonged in patients with ETI. The data also revealed a trend towards increased ETI with colder balloon temperatures in the LSPV. Further studies are warranted to evaluate these relationships, but the study suggest that it may be protective to keep total freeze time in the LIPV at or below 300 s and balloon temperatures in the LSPV at or above −51°C.

### 4.4 Incidence of gastroparesis

Interestingly, gastroparesis was found in 60% of patients at the time of EGD. There were no significant differences in the rate of gastroparesis between the two groups. Notably, no patients had clinical signs of persistent gastroparesis at follow‐up. Acute gastroparesis is a well‐known complication from CBA, but previously documented rates tend to be far lower.^19^, ^20^ Yakabe et al. studied a population of 269 patients who underwent CBA and found a rate of acute gastroparesis of 2.2%.^21^ Notably, in that study, gastroparesis was assessed for 2 days after ablation, after a minimum of a 15 h fast, and every case resolved within 4 weeks. The higher rate in the present study may be explained by the fact that the EGD was performed the day following ablation and therefore had a higher degree of post anesthesia effects. Further studies are warranted to better understand the nature of gastroparesis in the setting of CBA.

### 4.5 Effect on CBA efficacy

In regard to AF recurrence, the present study found no significant difference between the WRM and LET groups out to 12 months. This suggests that use of the esophageal heat‐exchange tube for esophageal warming does not have an effect on the efficacy of CBA, though additional studies would be necessary to confirm this finding.

### 4.6 Limitations

This study is limited by its population size since it was intended as a pilot study. It is also limited as a single‐center study. Though both the patient and gastroenterologist reviewing the EGDs were blinded, the operator was not blinded to the treatment arm. It is not likely that blinding of the operator would be possible. As with many studies examining risk of AEF, a surrogate marker (ETI) was used due to the low incidence of this complication. No AEF was identified despite these endoscopic ETI findings, so the implications for reduction of AEF are not clear based on these results. The diameter of the heat‐exchange tube is 1.2 cm, so it is possible that the device may not have consistent contact and coverage in a patient with a wide esophagus.

## 5 CONCLUSION

Use of the esophageal heat‐exchange tube for esophageal warming during CBA for AF was paradoxically associated with a higher risk of ETI. Further bench or animal studies may be beneficial to assess the use of different warming devices and parameters during ablation procedures using cryothermal energy. Additional strategies are needed to further reduce the risk of AEF during CBA.

## Data Availability

The data that support the findings of this study are available from the corresponding author upon reasonable request.
